# The Effect of Kinesiology Taping on the Hemiplegic Shoulder Pain: A Randomized Controlled Trial

**DOI:** 10.1155/2018/8346432

**Published:** 2018-12-10

**Authors:** Lin Yang, Jingyi Yang, Chengqi He

**Affiliations:** The Rehabilitation Department of the West China Hospital, Sichuan University, Chengdu, China

## Abstract

**Objective:**

The purpose of the study was to explore the effect of kinesiology taping on hemiplegic shoulder pain (HSP) in terms of pain intensity, magnitude of subluxation, muscle activity, and active range of motion (AROM).

**Design:**

Double-blind, placebo-controlled clinical trial.

**Setting:**

the Rehabilitation Center of the West China Hospital.

**Participants:**

Nineteen individuals suffering from HSP were recruited in this study.

**Intervention:**

Patients were randomly assigned into the taping group or control group. The taping group received therapeutic kinesiology taping and conventional treatment, while the control group received placebo taping (applied without tension) and conventional treatment.

**Main Outcome Measures:**

The shoulder pain intensity (numerical pain rating scale), magnitude of subluxation, muscle activity (measured by surface electromyography (sEMG)), and shoulder active range of movement (AROM) were assessed at the baseline, on the first day (immediately after taping) and 4 weeks after treatment (without taping).

**Results:**

All patients completed the trials. There were no significant differences between groups at the baseline. The taping group showed immediate improvement on the first day after taping in terms of pain intensity, magnitude of subluxation, and muscle activity (*p* < 0.05), whereas no significant changes seen in the control group (*p* > 0.05). After 4 weeks of treatment, the taping group showed significant changes in pain intensity, magnitude of subluxation, muscle activity, and AROM (*p* < 0.05). And significant differences in pain intensity and muscle activity could be seen between the two groups (*p* < 0.05).

**Conclusion:**

The results indicate that the kinesiology taping is effective in reducing the shoulder pain and subluxation and increasing muscle activity and AROM for patients with HSP after stroke.

## 1. Introduction

Shoulder pain is a common complication in hemiplegic patients after stroke. The incidence of hemiplegic shoulder pain (HSP) is reported to be approximately 17–72% [1–6]. HSP can inhibit the muscle contraction and limit efforts of patients to conduct exercise and delay the recovery in the motor function and influence capacity of performing daily activities and social participation [7–9]. This may further increase the length of a patient's stay in the hospital [10].

The mechanisms for HSP are not fully understood. Muscle weakness after stroke is believed to be a primary reason for HSP. As the shoulder muscles cannot contract effectively against gravity and external force during movement, the humeral head cannot be held in a proper position. Soft tissues around the shoulder may be gradually stretched and torn, resulting in HSP [11]. This understanding constitutes a reasonable mechanism to explain why soft tissue injuries are usually observed in HSP [12–15]. Other factors such as disturbed sensory and cognitive function are also proved to be involved in HSP [16].

Regardless of the limited understanding of the etiology of HSP, various therapeutic methods have been trialled to examine their effects on HSP. Different types of slings and strapping methods which can provide effective support to the shoulder are usually involved in the therapeutic strategy for HSP [6, 17]. Electrical therapy and appropriate exercise program are also proved to be necessary for the treatment of HSP [18–21]. However, evidence for the effectiveness of these conventional methods for HSP is limited. And some of these treatment methods have significant problems and limitations to their use, such as sling and strapping which may limit the movement of the shoulder and interfere with the recovery of the function [18, 21, 22].

Kinesiology taping, sometimes also called elastic taping, is a relatively new technique used in rehabilitation programs. This method is different from strapping or rigid taping technique. The elastic tape is a hypoallergenic skin tape with elastic components. This kind of elastic tape is designed to allow for a longitudinal stretch of 55–60% of its original extent. By using the kinesiology tape, different stretches and directions can yield different effects for reducing pain, correcting alignment, facilitating neuromuscular activity, and alleviating inflammation and edema [23–25]. Based on these notions about the features of kinesiology taping, it can be hypothesized that kinesiology taping can be used for patients with HSP to reduce the pain, correct the subluxation, activate the muscles of shoulder, and further restore the motor function. However, few studies have been done to provide evidence for the hypothesis. Therefore, the aim of this study is to investigate the effects of kinesiology taping on HSP.

## 2. Methods

### 2.1. Research Design

A double-blind, placebo-controlled clinical trial was carried out to investigate the effects of kinesiology taping on HSP. The blind method was used for the patients and therapists who provided conventional treatments and assessment. The primary outcome measurement was set as the pain intensity of the shoulder, and the secondary outcome measurements included the magnitude of subluxation, active range of motion (AROM), and muscle activity of the shoulder.

The study protocols were approved by the Ethics Committee of the West China Hospital of Sichuan University in China and were funded by the Sichuan Provincial Department of Public Health. And all participants provided written informed consent.

### 2.2. Participants

The trial was carried out in the Rehabilitation Center of the West China Hospital from April 2013 to September 2014. Nineteen participants with a diagnosed first-ever stroke were recruited to the study. All participants met the following inclusion criteria: (1) > 30 years of age; (2) period after stroke: >1 month and <6 months; (3) diagnosed as HSP with a period of more than 1 month, accompanied with shoulder subluxation; (4) adequate communication abilities; (5) the shoulder muscles can contract and move the shoulder more than 10 degrees but less than 90 degrees in flexion and/or abduction in sitting position, accompanying shoulder pain produced or increased; (6) normal light touch and pin-prick sensation on the affected shoulder; (7) the pain is caused by local problems: (1) manual palpation and movement can affect the pain and (2) reposition of the joint can relieve the pain.

The exclusion criteria include (1) history of serious conditions or diseases such as cancer; (2) skin problems, wounds, or infections on the affected shoulder; (3) skin allergy to the tape; (4) history of shoulder fracture on the affected side or history of shoulder sprain or subluxation before the stroke; (5) severe disease which may affect the study, such as uncontrolled hypertension or heart disease; (6) history of intra-articular steroid injection in the past 4 weeks.

### 2.3. Procedure

According to the recruited order and the corresponding random number sealed in an envelope, patients were assigned into the taping group or the control group. Before treatment, the demographic data and clinical characteristics of the participants were recorded. The outcome measures were assessed at the following time points: at the baseline (one day before the treatment), on the first day (immediately after taping on the first day, with taping applied), and after 4 weeks (on the following day after the 4 weeks treatment completion, without taping).

All the participants received treatment once a day, 5 days per week for 4 consecutive weeks. The treatment program included kinesiology taping, electrical therapy, and exercise treatment. Electrical therapy was applied first, followed by taping and finally exercise. The therapeutic taping was used for the taping group, while placebo taping (without tension) was applied for the control group.

Exercise therapy for the affected shoulder included passive and active assistant and active exercises. These exercises focused on restoring and improving the motor function of the shoulder. The range of motion (ROM) exercise of the affected shoulder was demanded to be less than 90 degrees to avoid potential injury to the joint. The exercise time for the affected shoulder was 20 min. The patients also received 40 mins exercise for the trunk and low limb.

Electric therapy (Bengao-computerized intermediate-frequency therapy apparatus, Beijing Bengao High technology Co. Ltd.) was applied in the study. The electrodes were placed over the posterior and anterior aspects of the shoulder. The pain-reduce program was chosen for all patients. The treatment time was set to 20 min, and the output current was set to 20–40 mA.

**Figure 1:**
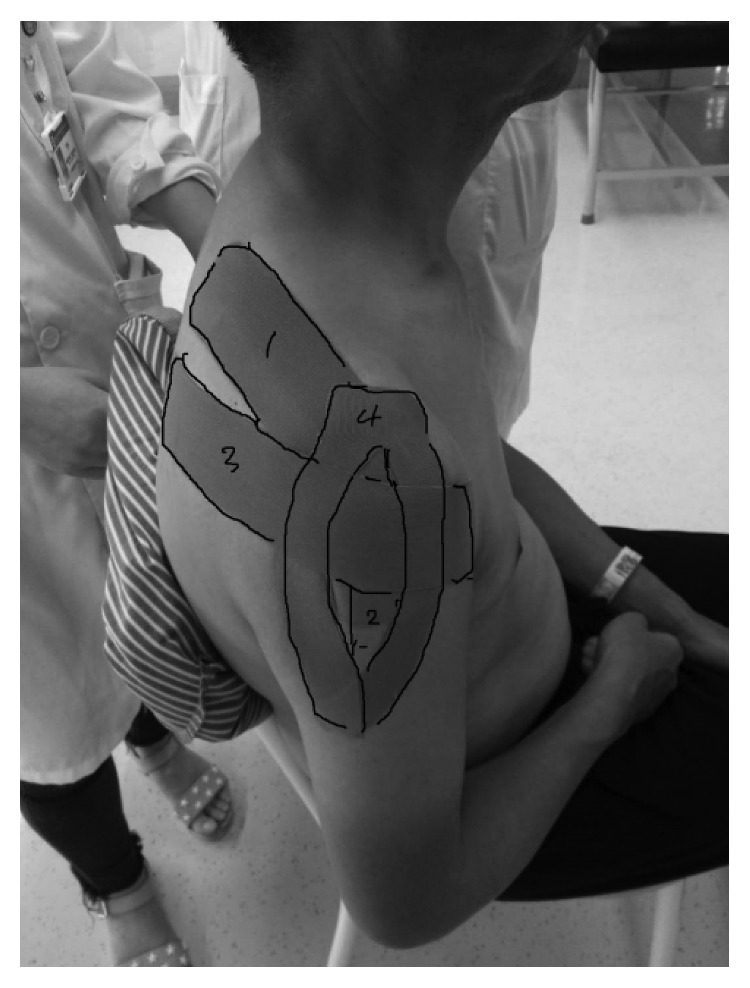
Kinesiology taping for patients. (1) supraspinatus; (2) middle part of deltoid; (3) teres minor; (4) anterior and posterior part of deltoid.

The therapeutic taping aimed to activate the neuromuscular function and produce mechanical support to the shoulder. Tapes of 5 cm width were used for patients. The taping method is shown in Figure 1. The facilitation technique was used for the deltoid, supraspinatus, and teres minor. First, the supraspinatus was taped. The shoulder was positioned in an abduction potion at about 30 degrees with a slight flexion and internal rotation, and the humeral head was repositioned to the normal place. The first 4 cm of the tape was applied to the original site of supraspinatus (superior medial border of the scapula) with no tension. Then, the remaining strip was applied over the muscle to the insertion site (greater tubercle of humerus) with about 25–50% of the full available tension. After this, the patient's shoulder was placed in abduction at 30 degrees. Taping of the middle part of deltoid muscle begun by attaching the first 4 cm of the strip over the acromion process with no stretch. Then, the rest of the strip was stretched downward to the deltoid tuberosity with 20–30% of tension. For taping the teres minor, the shoulder was flexed with a little internal flexion. The base of the tape was placed on the inferior angle of scapular. The rest of the strip was stretched with 15–25% of tension and placed along the axillary border of the scapula to the greater tuberosity of the humerus. The last one tape was used to reduce the subluxation of the shoulder and was cut into Y shape before taping. After reposition of the shoulder, the base of the tape was applied to the acromion process, and then, the two strips were stretched with a tension of 50–70% and placed along the anterior and posterior borders of deltoid separately to the deltoid tuberosity. For the placebo group, the tapes were applied on the same place but no tension was applied. The tapes were removed at about 8 pm. The time for taping was ensured to be 10 to 12 hours per day. After removing the tape, the skin was cleaned with warm water to keep it healthy.

### 2.4. Outcome Measures

#### 2.4.1. Pain Intensity

Shoulder pain was the primary outcome measure and was assessed by a numerical pain rating scale (NPRS) which has a high correlation coefficients with the visual analog scale and is simple to use [26]. The pain intensity is graded from 0 to 10 where 0 means no pain and 10 means the most intense pain. The maximum pain during shoulder flexing and abducting in sitting position was recorded.

#### 2.4.2. Magnitude of Shoulder Subluxation

The patient was kept unsupported in an upright posture without a backrest or armrests, with the arms in a neutral position hanging by the side of the body. The inferior border of the acromion and the highest of greater tubercle of the shoulder was palpated and marked. The vertical distance between the two points was measured with a tape measured and recorded. The difference of the distance between the affected and normal sides was defined as the magnitude of subluxation.

#### 2.4.3. AROM of the Shoulder

In a sitting position, the patient was instructed to first flex and then to abduct the affected shoulder as much as possible. The AROM of the affected shoulder in flexion and abduction was measured with the goniometer and recorded.

#### 2.4.4. Muscle Activity

Electrical sensors have emerged as an efficient way to characterize biomedical signals. The muscle activity was assessed with sEMG. The Muscle Tester ME6000 (Mega Electronics Ltd., Kuopio, Finland) was used for the purpose of the study. The muscle electrical activation of the middle part of deltoid and supraspinatus muscles during shoulder abducting in sitting position was recorded. Prior to the test, the skin was cleansed with 75% alcohol. Disposable 8 mm AgCI surface electrodes (Blue sensor, Ambu, Denmark) were attached over the middle part of deltoid and supraspinatus muscles on the affected side. Sampling of the muscle activity was done at a frequency of 1000 Hz. The data were downloaded to an EMG analyzer, Megawin 2.5 (Mega Electronics Ltd., Kuopio, Finland), where the data were analyzed. The average amplitude, which was presented with AEMG (*μ*V), was calculated for further analysis.

### 2.5. Data Analysis

Statistical analysis was carried out by using the SPSS version 20.0. The results were presented as mean (SD). The chi-square analysis was used to determine the group difference in sex and side of hemiplegia. Paired sample *t*-test and independent sample *t*-test were applied to examine the difference within group and between the two groups, respectively. The significance level was set at *p* < 0.05.

## 3. Results

Totally, 19 patients were recruited in the study. The demographic characteristics and measurements at the baseline are shown Table 1. There were no significant differences between the two groups at the baseline. All the participants completed the whole trial, and there were no side effect observed.

Based on the data listed in Tables 2–5, the taping group showed an immediate and significant improvement in pain intensity, magnitude of subluxation, and AEMG on the first day immediately after taping, compared with the baseline (*p* < 0.05), whereas there was no significant changes in the control groups in these outcomes. Both groups showed no significant changes in the AROM in flexion and abduction on the first day (*p* > 0.05). After 4 weeks of treatment, significant improvement was seen in the taping group in terms of pain intensity, magnitude of subluxation, AEMG, and AROM (both flexion and abduction). Based on the baseline, the amount of improvement in the pain intensity, magnitude of subluxation, AEMG, and AROM were significantly greater than the control group (*p* < 0.05).

The statistical power was calculated by using the software G^∗^Power. The calculation was based on the primary measure, NPRS. The power is 0.99 which indicates that power level is acceptable, and the sample size is enough for this study.

## 4. Discussion

To our knowledge, this is the first study aimed at exploring the effects of kinesiology taping on HSP in terms of pain, shoulder subluxation, AROM, and muscle activity of the shoulder. The results indicated that kinesiology taping is an effective method for HSP management.

It has been noticed that some other types of taping methods were trialled to examine their effect on HSP. Despite the difference in tapes and taping methods, most of these studies focused on the effect of taping on preventing the development of HSP [27, 28]. As far as we know, few studies examined the effectiveness of kinesiology taping in treating HSP.

Actually, kinesiology taping has been widely used to treat musculoskeletal problems. Its effects on nonspecific pain, such as neck pain, impinged shoulder pain, and knee pain, have also been examined. In the present study, a significant reduction of pain (NPRS, from 4.3 points to 0.3 points, *p* < 0.05) was observed immediately after taping was applied on the first day. The immediate effectiveness of kinesiology taping in modulating pain was reported in previous studies focusing on treating impinged shoulder pain, neck pain, and low back pain [25, 29–31]. However, the mechanism for the immediate effect is still unknown. One of the proposed mechanisms suggests increasing the afferent feedback to the spine. Under the gate control theory, then increase in afferent stimulus can reduce the conducting of nociception into the central nervous system [32]. In addition, the effect of alignment correction may be another potential mechanism. The alignment correction effect on skin and shoulder posture has been proven [33, 34]. The present study also showed notable effectiveness in reducing the shoulder subluxation. It is reasonable that the reduced subluxation can decrease the stimulation to nociception sensors, resulting in pain modulation. Different from the immediate effect on pain modulation, the reduced pain observed after 4 weeks could be ascribed to healing because when the assessment at week 4 posttreatment was done without taping.

Besides the reduction in pain intensity, kinesiology taping also yielded notable decrease of subluxation. Even though the reliability and validity of the method for assessing the subluxation have not been examined, considering the blind method was applied to the assessor and patients, the results have credibility for discussion. The alignment correction effect of taping has been widely examined and accepted [34, 35]. Reducing the subluxation can provide an opportunity to injured tissue to heal. The effectiveness observed after 4 weeks can be ascribed to the enhanced healing process. Based on the finding, it can be concluded that kinesiology taping can enhance the healing of injured tissue around the shoulder. Similarly, a sling can also provide mechanical support to the subluxed shoulder [36]. Previous studies indicated that a sling is able to bring benefit to patients with HSP in shoulder subluxation [36] and walking efficiency [37]. However, a sling may limit the movement of the shoulder. Most importantly, the sling usually places the arm anteriorly. This may aggravate the flexion pattern of upper extremity and increase the scapular lateral rotation pattern which has been proved to be linked with HSP [38]. This may be one of the reasons why sling treatment leaded to a conflicting result in previous studies [18]. Strapping may be another alternative to provide support to the shoulder. However, the rigid tape cannot work effectively during movement. A study demonstrated that 15–20 min of exercise can cause the rigid tape losing its function to restrict joint [39]. In contrast with strapping, kinesiology taping can provide effective support to the shoulder. Its elastic quality conforms to the body and allows for movement.

In addition, the muscle activation effect of kinesiology taping has been discussed and studied. Macgregor et al. designed a study to investigate the effect of taping on muscle activity in people with patellofemoral pain. Results demonstrated that stretching to the skin via taping can increase muscle activity [40]. Some other recent studies also provided evidence suggesting that taping can affect the muscle activity [41, 42]. However, the muscle activation effect of taping was not supported by the study conducted by Ryan and Rowe in which the symptomatic participants did not show significant changes in surface electromyography indices after taping [43]. In the present study, facilitation technique has been applied to the middle part of deltoid, supraspinatus, and teres minor. Data showed significant improvement AEMG in the taping group after taping, implying that kinesiology taping can activate the muscles. However, the increased AEMG may also be contributed to the reduced inhibition of pain to muscles and alternated kinesiology induced by increased subluxation. The muscle activation effect might therefore be a result of multiple mechanisms.

Despite the immediate improvement in pain, subluxation, and muscle activity, no significant changes in AROM were observed after tapping. It is out of our expectation that, along with the significant reduction of pain, the AROM shoulder increased correspondingly. It is known that pain is an inhibiting factor to the neuromuscular activity [7] and the effort of a patient to move. The results might indicate that muscle weakness was a dominant reason for the limited AROM. After 4 weeks of treatment, the taping group showed much greater improvement in AROM than the control group, indicating that kinesiology taping can enhance the recovery of motor function when accompanied with conventional treatment. The effectiveness might be contributed to the reduction of pain which can enhance the initiative of patient to conduct exercise. Furthermore, kinesiology taping seems to be able to activate the neuromuscular function, which is very crucial for the recovery of the motor function in patients after stroke.

### 4.1. Study Limitations

There were some limitations in the present study that should be noted. First, the characteristics of the patients in this study met specific criteria. For example, only patients whose pain can be reduced by joint reposition were recruited. Second, there was no special examination, such as X-ray or ultrasound, adopted in the study to document the shoulder injury and healing. Third, the sample size was small, and all participants belonged to the same hospital. Finally, only 4 weeks of treatment was conducted, without long-term follow-up data. Hence, the ability to generalize the findings in the present study to the poststroke population is limited.

## 5. Conclusion

The present study revealed the effectiveness of kinesiology taping for HSP. Kinesiology taping might be a good alternative for relieving shoulder pain, improving the AROM, subluxation, and muscle activity of the shoulder in patients with HSP after stroke.

## Figures and Tables

**Table 1:** Baseline characters of patients in the two groups.

	Taping group	Control group	*p* value
Number	10	9	
Age (years)	59 (3.2)	60 (2.3)	0.939
Male : female	7 : 3	6 : 3	0.875
Left : right (hemiplegia)	4 : 6	5 : 4	0.498
Time poststroke (weeks)	18.3 (0.82)	19.2 (2.49)	0.502
Pain	4.3 (1.2)	5.0 (0.7)	0.111
Subluxation (cm)	1.22 (0.39)	1.18 (0.33)	0.807
ROM (F) (degrees)	27.8 (6.1)	31.7 (7.6)	0.238
ROM (Abd.) (degrees)	24.8 (5.5)	25.6 (6.8)	0.763
AEMG (S) (*μ*V)	152.6 (27.9)	163.3 (33.1)	0.925
AEMG (D) (*μ*V)	126.8 (11.5)	134.6 (10.4)	0.482

*Note.* F = flexion; Abd. = abduction; S = supraspinatus muscle; D = deltoid muscle.

**Table 2:** Pain intensity assessed at different time points.

	*n*	Baseline	First day	4 weeks later
Taping group	10	4.3 (1.2)	0.6 (0.6)^*∗*∧^	1.4 (0.7)^*∗*∧^
Placebo group	9	5.0 (0.7)	4.8 (0.8)^*∗*^	3.4 (0.8)^*∗*∧^
*p* value		0.111	0.000	0.000

^*∗*^Independent samples *t-*test, compared between groups, *p* < 0.05; ^*∧*^paired sample *t-*test, compared between before and after treatment, *p* < 0.05.

**Table 3:** Magnitude of subluxation (cm) of the shoulder.

	Baseline	First day	4 weeks later
Taping group	1.22 (0.39)	0.71(0.20)^*∗*∧^	0.91 (0.31)^*∧*^
Control group	1.18 (0.33)	1.18 (0.31)^*∗*^	1.18 (0.26)
*p*	0.807	0.01	0.062

^*∗*^Independent samples *t-*test, compared between the taping group and the control group, *p* < 0.05; ^*∧*^paired sample *t-*test, compared between before and after treatment, *p* < 0.05.

**Table 4:** The AEMG (*μ*V) tested at different time points.

Group	Muscles	AEMG (*μ*V)		
		Baseline	First day	4 weeks later
Taping group	Deltoid	126.8 (11.5)	140.0 (13.9)^*∧*^	221.6 (34.7)^*∗*∧^
	Supraspinatus	152.6 (27.9)	177.0 (33.1)^*∧*^	273.3(37.7)^*∗*∧^

Control group	Deltoid	134.6 (10.4)	135.7 (12.9)	167.78 (10.2)^*∗*∧^
	Supraspinatus	163.3 (33.1)	161.3 (35.6)	198.1 (33.7)^*∗*∧^

^*∗*^Independent samples *t-*test, compared between the taping group and the control group, *p* < 0.05; ^*∧*^paired sample *t-*test, compared between before and after treatment, *p* < 0.05.

**Table 5:** The AROM of the shoulder (degree) assessed at different time points.

Group		Baseline	First day	4 weeks later
Taping group	Flexion	27.8 (6.1)	28.8 (6.2)	40.1 (6.8)^*∧*^
	Abduction	24.8 (5.5)	26.7 (5.4)^*∧*^	33.4 (5.1)^*∧*^

Control group	Flexion	31.7 (7.7)	31.7 (7.7)	37.9 (9.6)^*∧*^
	Abduction	25.6 (6.8)	26.0 (6.9)	29.8 (9.2)^*∧*^

^*∗*^Independent samples *t*-test, compared between the taping group and the control group, *p* < 0.05; ^*∧*^paired sample *t*-test, compared between before and after treatment, *p* < 0.05.

## Abbreviations

HSP: Hemiplegic shoulder pain
AROM: Active range of movement
sEMG: Surface electromyography
NPRS: Numerical pain rating scale.
